# Oral health and longitudinal changes in fasting glucose levels: A nationwide cohort study

**DOI:** 10.1371/journal.pone.0253769

**Published:** 2021-06-29

**Authors:** Tae-Jin Song, Yoonkyung Chang, Jimin Jeon, Jinkwon Kim

**Affiliations:** 1 Department of Neurology, Seoul Hospital, Ewha Womans University College of Medicine, Seoul, Republic of Korea; 2 Department of Neurology, Mokdong Hospital, Ewha Womans University College of Medicine, Seoul, Republic of Korea; 3 Department of Neurology, Yongin Severance Hospital, Yonsei University College of Medicine, Yongin, Republic of Korea; University of the Pacific, UNITED STATES

## Abstract

We investigated the association between oral hygiene indicators of periodontitis, tooth loss, and tooth brushing on the longitudinal fasting glucose level in non-diabetic subjects. Using a nationwide health screening database in Korea, we included non-diabetic individuals who received a health screening program with oral health check in 2009–2010. We constructed a linear mixed model for the longitudinal data of fasting glucose from the baseline to 2015. During the 4.84-year of median follow-up, 91,963 individuals (mean age 56.2 at baseline) underwent 392,780 health examinations with fasting glucose level (mmol/L). The presence of periodontitis was 39.3%. In the multivariate linear mixed analysis, periodontitis was related with increased fasting glucose levels (β = 0.0084, standard error = 0.0035, *p* = 0.018). Similarly, tooth loss was associated with increased level of fasting glucose (β = 0.0246, standard error = 0.0038, *p* < 0.001). Compared with tooth brushing ≤2 times/day, tooth brushing ≥3 times/day was associated with decreased fasting glucose levels (β = -0.0207, standard error = 0.0033, *p* < 0.001). Our data showed that periodontitis and tooth loss were associated with increased fasting glucose levels in non-diabetic individuals. The study findings imply that frequent tooth brushing may reduce fasting glucose levels. Further research is needed to determine the effect of periodontal intervention on glycemic control.

## Introduction

Diabetes and hyperglycemia are major global health problems that pose a high risk of macrovascular and microvascular complications [1,2]. Over time, hyperglycemia leads to severe damage and dysfunction of different organs and tissues, including the heart, blood vessels, kidneys, eyes, and nerves [3]. Currently, ensuring optimal glycemic control is the primary therapeutic target for the prevention of diabetic complications [4]. However, inadequate glycemic control is prevalent in clinical practice; thus, there is an urgent need for effective treatment strategies to improve glycemic control [4].

Periodontitis is a common and chronic inflammatory disease in humans [5]. It refers to inflammatory reactions involving the tissues that surround teeth, such as the alveolar bone, gingiva, and periodontal ligaments [6]. Along with periodontitis, dental caries, tooth loss, and tooth brushing frequency are considered as oral hygiene indicators [7–9]. Periodontitis and poor oral hygiene may cause local infection, inflammation, and systemic inflammatory responses [6]. The inflammatory response is closely related to endothelial dysfunction and insulin resistance, a major mechanism of hyperglycemia and diabetes [10,11]. The close association between inflammatory biomarkers and hyperglycemia has been confirmed in previous epidemiologic studies [12]. However, there is insufficient information on the relationship between oral hygiene practices and longitudinal changes in glucose levels. We hypothesized that periodontitis and poor oral hygiene is associated with increased fasting glucose levels and that good oral hygiene, such as frequent tooth brushings, is related to reduced fasting glucose levels in a long-term study. We aimed to test this hypothesis using a nationwide population-based health screening database with an oral health examination in Korea.

## Materials and methods

### Source of data

The current study was retrospectively performed using the National Health Insurance Service-National Health Screening Cohort (NHIS-HEALS) database [13]. The NHIS in Korea conducts a free nationwide health screening program for the whole population over 40 years old every two years. NHIS-HEALS contains data of health screening program with oral health check, laboratory test, including measurement of fasting glucose, blood pressure, body mass index (BMI), and lifestyle questionnaire. The fasting glucose level was measured using a venous blood sample in a fasting state (>6-hour). Since the NHIS is a single payment institution, the NHIS-HEALS included health claims data of hospital visits, admission, medical procedure, drug prescription, and diagnosis. At each hospital visit, a diagnostic code was recorded based on the International Statistical Classification of Diseases and Related Health Problems, 10^th^ revision (ICD-10). Due to the retrospective nature and analysis with the fully anonymized data from NHIS-HEALS, this study was approved by the Institutional Review Board of our institution (Ewha Womans University Medical Center 2020-08-018). Informed consent was waived, and all investigation was performed according to the Declaration of Helsinki 2013.

### Study participants

**Fig 1:**
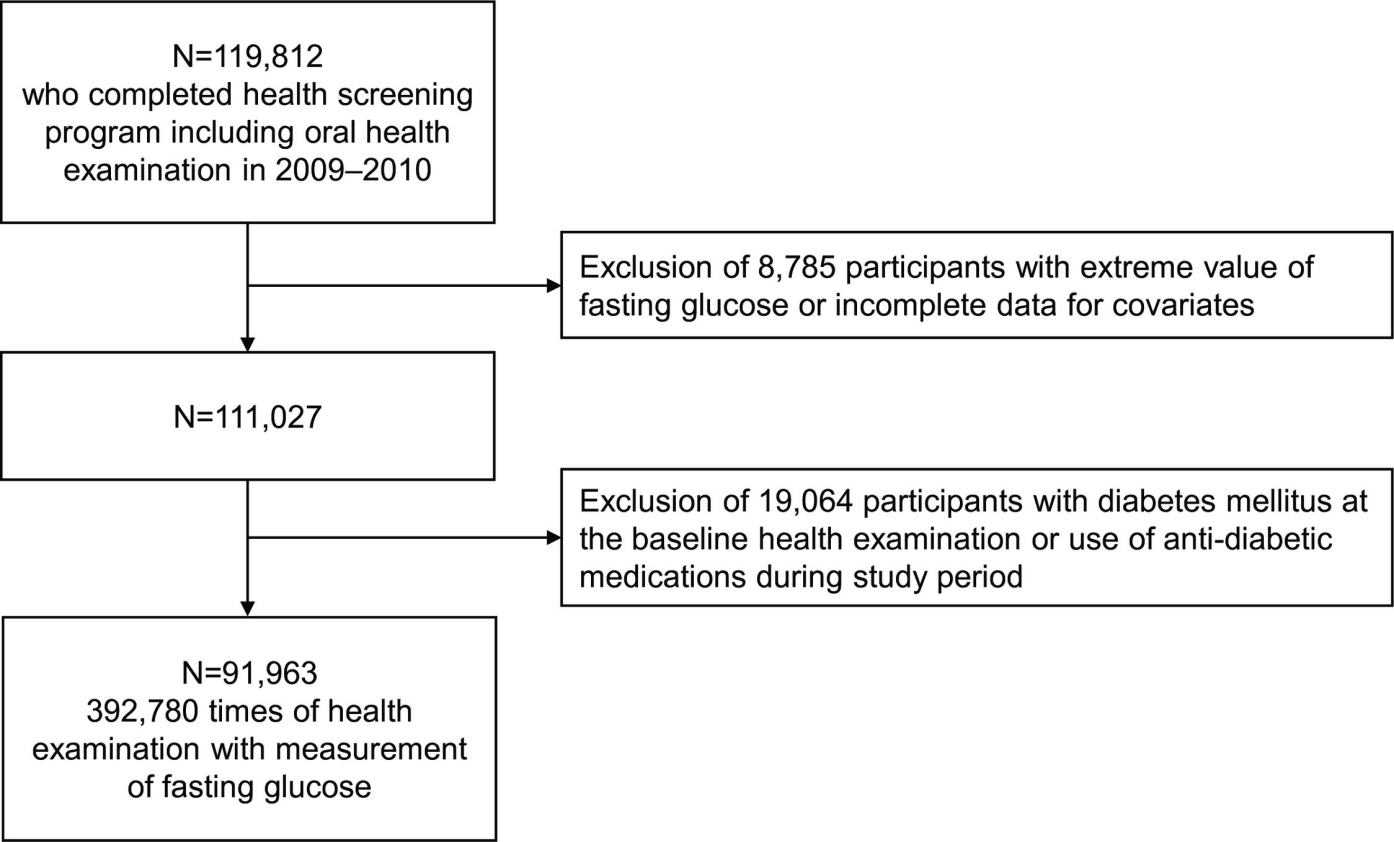
Flowchart of patient inclusion and exclusion.

From the NHIS-HEALS database, we included only non-diabetic individuals who participated in the national health screening program, including oral health check in 2009–2010 (baseline examination). The exclusion criteria were: 1) extreme value of fasting glucose level (≥15 mmol/L or ≤3 mmol/L) which may be result from wrong measurement, 2) incomplete data for at least one of the covariates, 3) diagnosis of diabetes at or before the baseline, and 4) use of any antidiabetic medications (sulfonylurea, biguanide, thiazolidinedione, alpha-glucosidase inhibitor, dipeptidyl peptidase-4 inhibitor, sodium-glucose cotransporter-2 inhibitors, glucagon-like peptide-1 receptor agonist, and insulin) during the study period (to exclude confounding effect of the antidiabetic medications on the fasting glucose). Finally, we included 91,953 non-diabetic individuals and 392,793 measurements of fasting glucose from the serial health screening program (Fig 1).

### Data collection

At the baseline health examination, the presence of periodontitis, dental caries, and tooth loss was evaluated by a dentist.[14,15]. The presence of periodontitis was determined as when the following ICD-10 codes [acute periodontitis (K052), chronic periodontitis (K053), and periodontitis (K054)] were claimed more than once (≥ 2 times) by a dentist or when the treatment codes were claimed [health claim codes: U1010 (subgingival curettage), U1020 (new attachment excision), U1051-1052 (periodontal flap), U1071-1072 (bone graft for alveolar bone defects), U1081-1083 (tissue regeneration)] with the relevant ICD-10 codes (K052, K053, K054) by a dentist within the last year or when community periodontal index on the index teeth (11, 16, 17, 26, 27, 31, 36, 37, 46, and 47) at least once (gingival bleeding at probing) in the oral health examination [14,15]. The frequency of tooth brushing was dichotomized into ≤2 times per day and ≥3 times per day based on a self-questionnaire.

The collected data included demographics (sex, age, household income), the presence of hypertension, and chronic kidney disease (CKD) at the baseline examination. The presence of hypertension was determined if a subscriber had health claims for prescription of antihypertensive medications with diagnostic codes of hypertension (I10–15), systolic blood pressure ≥140 mmHg, diastolic blood pressure ≥90 mmHg, or reported having a hypertension in the survey. The presence of CKD was evaluated based on the diagnostic codes (N18, N18.1–18.5, N18.9) or an estimated glomerular filtration rate of <60 mL/min/1.73 m^2^ [16].

Data on the fasting glucose level, BMI, and the lifestyle questionnaire were obtained from serial health examinations in NHIS-HEAL from the baseline to 2015. The lifestyle questionnaire included questions of smoking status (never, former, current smoker), alcohol consumption frequency (average frequency per week: none, 1–2 times, 3–4 times, ≥5 times), and workout frequency (average frequency per week regardless of the intensity or duration of the exercise: none, 1–4 days, ≥5 days) [17,18].

### Statistical analysis

Differences between groups were compared using the chi-square test for categorical variables and the independent t-test or analysis of variance for continuous variables, respectively. To evaluate whether oral health markers were associated with longitudinal fasting glucose levels, we constructed a linear mixed model. In the linear mixed analysis, the fixed effects were sex, age at baseline, oral health indicators, household income, presence of hypertension, CKD, and data from the serial health examinations (BMI, alcohol consumption, smoking, and workout frequency). Manipulation of data and the statistical analysis were performed using SAS software, 9.4 version (SAS Inc., Cary, NC, USA) and R software, 3.3.3 version (R Foundation for Statistical Computing, Vienna, Austria; http://www.r-project.org/). A two-tailed *p*-value of <0.05 was considered to be statistically significant.

## Results

### Characteristics

According to the inclusion and exclusion criteria, 91,963 non-diabetic individuals who competed in an oral health check in 2009–2010 were finally included (Fig 1). There were 392,780 times of fasting glucose measurements during the median follow-up of 4.84 years (interquartile range, 4.08–5.95 years). The median number of fasting glucose measurements per participant was 4 (interquartile range, 3–6).

The baseline characteristics of the participants are summarized in Table 1. Men accounted for 58.0% of the participants, and the mean age was 56.16 ± 7.60 years at baseline. The mean ± standard deviation of baseline fasting glucose level was 5.23 ± 0.60 mmol/L. The prevalence of periodontitis, tooth loss, and dental caries were 39.3%, 24.9%, and 51.2%, respectively. The proportion of those who performed tooth brushing at least three times per day was 50.1%. The characteristics of the participants according to the presence of periodontitis are shown in Table 2. The baseline fasting glucose level was high in participants with periodontitis compared to those without periodontitis.

**Table 1:** Clinical characteristics of the study participants at the baseline health examination.

Variable	Values
Sex, male	53308 (58.0)
Age, year	56.16 ± 7.60
Household income	
Q1, lowest	21988 (23.9)
Q2	21501 (23.4)
Q3	26151 (28.4)
Q4, highest	22323 (24.3)
Smoking status	
Never	49124 (53.4)
Former	26391 (28.7)
Current	16448 (17.9)
Alcohol consumption, frequency per week on average	
None	51149 (55.6)
1–2 times	28466 (31.0)
3–4 times	8755 (9.5)
≥5 times	3593 (3.9)
Exercise, days per week on average	
None	19991 (21.7)
1–4 days	45133 (49.1)
≥5 days	26839 (29.2)
Anthropometric measurements	
Body mass index, kg/m^2^	23.83 ± 2.76
Comorbidities	
Hypertension	33250 (36.2)
Chronic kidney disease	8410 (9.1)
Fasting glucose level (mmol/L)	5.23 ± 0.60
Oral health marker	
Periodontitis	36149 (39.3)
Dental caries	47095 (51.2)
Tooth loss	22856 (24.9)
Number of tooth brushing per day	
0–2 times	45895 (49.9)
≥3 times	46068 (50.1)

Data are expressed as the mean ± standard deviation or n (%). Q: Quartile.

**Table 2:** Clinical characteristics of the study participants according to the presence of periodontitis.

Variable	Periodontitis (-) N = 55814	Periodontitis (+) N = 36149
Sex, male	31594 (56.6)	21714 (60.1)
Age, year	55.93 ± 7.58	56.50 ± 7.62
Household income		
Q1, lowest	13034 (23.4)	8954 (24.8)
Q2	12809 (22.9)	8692 (24.0)
Q3	15920 (28.5)	10231 (28.3)
Q4, highest	14051 (25.2)	8272 (22.9)
Smoking status		
Never	30577 (54.8)	18547 (51.3)
Former	15964 (28.6)	10427 (28.8)
Current	9273 (16.6)	7175 (19.8)
Alcohol consumption, frequency per week on average		
none	31546 (56.5)	19603 (54.2)
1–2 times	17235 (30.9)	11231 (31.1)
3–4 times	5104 (9.1)	3651 (10.1)
≥5 times	1929 (3.5)	1664 (4.6)
Exercise, days per week on average		
none	12093 (21.7)	7898 (21.6)
1–4 days	27643 (49.5)	17490 (48.4)
≥5 days	16078 (28.8)	10761 (29.8)
Anthropometric measurements		
Body mass index, kg/m^2^	23.80 ± 2.76	23.87 ± 2.76
Comorbidities		
Hypertension	19879 (35.6)	13371 (37.0)
Chronic kidney disease	5502 (9.9)	2908 (8.0)
Fasting glucose level (mmol/L)	5.22 ± 0.60	5.24 ± 0.60
Oral health marker		
Dental caries	21411 (38.4)	25684 (71.1)
Tooth loss	11900 (21.3)	10956 (30.3)
Number of tooth brushing per day		
0–2 times	27277 (48.9)	18618 (51.5)
≥3 times	28537 (51.1)	17531 (48.5)

Data are expressed as the mean ± standard deviation or n (%). Q: Quartile.

### Periodontitis and oral hygiene indicators with longitudinal fasting glucose levels

**Fig 2:**
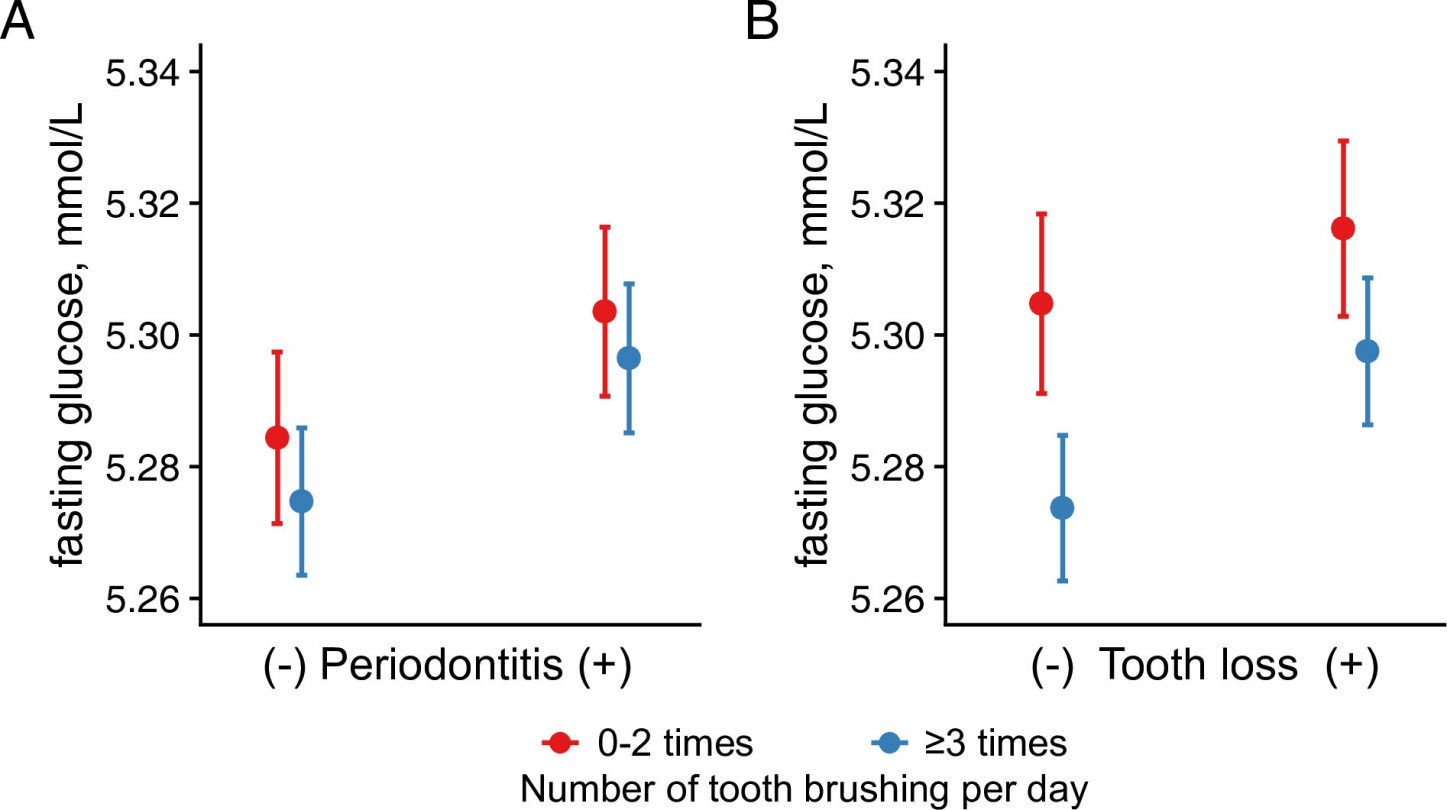
Fasting glucose levels according to the number of tooth brushing considering the periodontitis and tooth loss. Data are presented as mean and 95% confidence intervals of fasting glucose level derived from multivariable linear mixed models considering the interactions. There is no significant interaction effect (A: *p* for tooth brushing * periodontitis = 0.701, B: *p* for tooth brushing * tooth loss = 0.101).

In the linear mixed analysis for the longitudinal level of fasting glucose (Table 3), we found a significant positive association between periodontitis and fasting glucose levels (β = 0.0084 mmol/L, standard error [SE] = 0.0035, *p* = 0.018). Tooth loss was also significantly associated with increased fasting glucose levels (β = 0.0246 mmol/L, SE = 0.0038, *p* < 0.001). However, dental caries was not significantly associated with fasting glucose levels (β = 0.0053 mmol/L, SE = 0.0035, *p* = 0.122). Compared with tooth brushing ≤2 times per day, tooth brushing ≥3 times per day was significantly associated with decreased fasting glucose levels (β = -0.0207 mmol/L, SE = 0.0033, *p* < 0.001). To evaluate the potential interactions between the markers of poor oral hygiene and tooth brushing, we constructed mixed models for fasting glucose levels, including the interaction terms between the variables (Fig 2). There was no interaction between periodontitis and the number of tooth brushing (*p* = 0.701) and between tooth loss and the number of tooth brushing (*p* = 0.101).

**Table 3:** Association between oral hygiene markers and fasting glucose levels.

	Fasting glucose level, mmol/L		
	Beta	Standard error	*p*-value
Oral health marker			
Periodontitis	0.0084	0.0035	0.018
Dental caries	0.0053	0.0035	0.122
Tooth loss	0.0246	0.0038	<0.001
Oral hygiene care			
Number of tooth brushing per day			
0–2 times	Ref		
≥3 times	-0.0207	0.0033	<0.001

Data are derived from multivariable mixed model analysis for fasting glucose level, with sex, age at baseline, time from the baseline examination, household income, body mass index, smoking status, alcohol consumption, exercise, body mass index, presence of hypertension, presence of chronic kidney disease, and markers for oral health indicators as fixed effects variables.

## Discussion

The key findings of the current study include the following: 1) markers of poor oral hygiene, including periodontitis and tooth loss, were positively associated with hyperglycemia, and 2) frequent tooth brushing may decrease fasting glucose levels. Achieving glycemic control helps prevent and delay the development of diabetes and diabetic complications [19]. To regulate fasting glucose levels, a healthy diet and body weight and regular exercise are strongly recommended [20]. Despite these multifactorial approaches, glycemic control is often insufficient, posing a clinical and public health challenge [21]. Our data suggest that poor oral hygiene is a potential therapeutic target for glycemic control, implying that interventions to improve oral hygiene, including frequent tooth brushing, may improve long-term fasting glucose regulation.

Although our study was not able to evaluate disease mechanisms, the following hypotheses may help explain the study findings. Periodontitis or poor oral hygiene is associated with chronic inflammatory reactions, leading to inflammatory responses in the teeth and associated supporting tissues. Tissue injury due to tooth loss or periodontitis enables oral microorganisms to enter the systemic circulation [22]. This oral cavity biofilm dysbiosis can activate pathways related to inflammatory cytokines [22,23]. Lipopolysaccharide (LPS), a component of the cell wall of oral bacteria, can induce innate immune responses. Further, the activity of LPS is increased in diabetes [24,25]. Individuals with poor oral hygiene have elevated levels of systemic inflammatory biomarkers [23,26]. Increase in the levels of inflammatory markers is positively associated with insulin resistance, which in turn increases the risk of new-onset diabetes [27,28]. Therefore, inflammatory reactions due to poor oral hygiene can predispose an individual to impaired glycemic control and hyperglycemia.

Numeric evidence suggests a two-way (bidirectional) relationship of periodontitis with diabetes; diabetes increases the risk of periodontitis, and periodontal inflammation leads to poor glycemic control and insulin resistance [29,30]. In a Japanese community-based study, severe periodontal diseases compared with relatively less severe periodontal diseases were significantly associated with impaired glucose tolerance [31]. The risk of periodontal disease in poorly controlled diabetes was 2.9 times higher than that in individuals without diabetes [32]. In a previous study performed in Taiwan, which defined periodontitis based on the ICD-9 code, the risk of periodontitis was higher in diabetes than non-diabetes [33]. Similarly, in the European National Health Survey, diabetes has an increased likelihood of periodontal disease compared with non-diabetes controls (adjusted odds ratio of periodontitis in diabetes was 1.22) [34]. A recent meta-analysis has also shown consistent results regarding the positive association between periodontitis and hyperglycemia [35]. As with periodontitis, there have been prior reports of a close relationship between tooth loss and diabetes [36,37]. In the US population, tooth loss was approximately two times higher in patients with hyperglycemia than in individuals without hyperglycemia [38]. Our results are consistent with prior studies’ findings and provide further emphasis on the significant association between poor oral hygiene and increased fasting glucose levels.

If the periodontal inflammatory reaction contribute the development of diabetes, frequent tooth brushing may reduce risk of diabetes mellitus [15]. In a Japanese case-control study, less frequent tooth brushing had an approximately 1.6 times higher risk of diabetes [39]. Diabetic patients who brushed their teeth frequently were more likely to achieve appropriate glycemic control than those who brushed their teeth less frequently [40]. Our findings added evidence for the negative relationship between tooth brushing and longitudinal glucose levels based on the population-based health examination data. There is increasing evidence to suggest that frequent tooth brushing is an effective intervention in reducing both periodontal inflammation and the risk of heart failure, stroke, gastrointestinal cancer, and new-onset diabetes [15–18,41–43].

In the current study, dental caries was not associated with fasting glucose levels. However, previous studies have reported a positive association between dental caries and hyperglycemia or diabetes [44,45]. This disparity may be owing to differences in the study design or population. Moreover, we could not investigate the dose-related response because our data lacked information on the number or severity of dental caries. Therefore, more research is needed to elucidate the relationship between dental caries and fasting glucose levels.

Our study has several limitations. First, the current study involved only Korean participants; hence, caution is needed while generalizing the study results to other races. Second, periodontitis diagnosis based on ICD-10 codes does not fully cover the recently published definition of periodontal disease [46]. Third, detailed information on periodontitis (probing depths, bleeding after probing procedure, and attachment loss), number of missing teeth, causes of tooth loss, and severity of dental caries were not available from the oral health examination data. Fourth, this study used a longitudinal observational design without interventions; thus, we could not establish a causal relationship between hyperglycemia and oral hygiene.

In conclusion, periodontitis and tooth loss were positively associated with hyperglycemia, while frequent tooth brushing was negatively related to fasting glucose levels. Improving oral hygiene may be a plausible intervention to achieve adequate glycemic control.
